# Hyperactive TGF-β Signaling in Smooth Muscle Cells Exposed to HIV-protein(s) and Cocaine: Role in Pulmonary Vasculopathy

**DOI:** 10.1038/s41598-017-10438-3

**Published:** 2017-09-05

**Authors:** Pranjali Dalvi, Himanshu Sharma, Tomara Konstantinova, Miles Sanderson, Amy O’ Brien-Ladner, Navneet K. Dhillon

**Affiliations:** 1 Division of Pulmonary and Critical Care Medicine, Department of Medicine, University of Kansas Medical Center, Kansas City, Kansas USA; 2 Department of Molecular & Integrative Physiology, University of Kansas Medical Center, Kansas City, Kansas USA

## Abstract

We earlier demonstrated synergistic increase in the proliferation of pulmonary smooth muscle cells on exposure to HIV-proteins and/or cocaine due to severe down-modulation of bone morphogenetic protein receptor (BMPR) axis: the anti-proliferative arm of TGF-β super family of receptors. Here, now we demonstrate the effect of HIV-Tat and cocaine on the proliferative TGF-β signaling cascade. We observed a significant increase in the secretion of TGF-β1 ligand along with enhanced protein expression of TGFβ Receptor (TGFβR)-1, TGFβR-2 and phosphorylated SMAD2/3 in human pulmonary arterial smooth muscle cells on treatment with cocaine and Tat. Further, we noticed an increase in the levels of p-TAK1 complexed with TGFβR-2. Concomitant to this a significant increase in the activation of TAK1-mediated, SMAD-independent downstream signaling molecules: p-MKK4 and p-JNK was observed. However, activation of MKK3/6-p38MAPK, another axis downstream of TAK1 was found to be reduced due to attenuation in the protein levels of BMPR2. Both SMAD and non-SMAD dependent TGFβR cascades were found to contribute to hyper-proliferation. Finally the increase in the levels of phosphorylated TGFβR1 and TGFβR2 on exposure to HIV-proteins and cocaine was confirmed in pulmonary smooth muscle cells from cocaine injected HIV-transgenic rats and in total lung extracts from HIV infected cocaine and/or opioid users.

## Introduction

The Human immunodeficiency virus-1 (HIV-1) pandemic has spread to infect an estimated 40 million individuals worldwide. Highly active antiretroviral therapy (HAART) has been effective in curbing the infectious complications and also increasing the life-span of HIV patients. Ironically, this extended life span has resulted in notable increased incidence of non-infectious complications such as pulmonary arterial hypertension (PAH). Many echocardiography studies indicate elevated pulmonary artery pressures in HIV-infected asymptomatic individulas^1, 2^. Recent studies report prevalence of pulmonary hypertension in 2.6% to 7.7% of HIV-infected asymptomatic patients^3–6^. Nevertheless, the burden of HIV related PAH (HRPAH) in developing countries like Africa with approximately 23 million of people infected with HIV-1 could be devastating, with an estimated prevalence of HRPAH to be 5% according to one study^7^. Furthermore, recent report by Quezada *et al*. suggests 10% prevalence of HRPAH in the cohort from Spain^8^. Compared to other forms of PAH, mortality is the highest in PAH associated with HIV-infection^9^ with minimal improvement in hemodynamics with currently available therapies. Importantly, studies have shown that intravenous drug use (IVDU) is one of the leading risk factors in the development of HRPAH^5, 8, 10, 11^ with common occurrence among cocaine users.

The underlying cause of PAH is still unknown, although it has been found that vascular remodeling contributes to the development of PAH through increased pulmonary vascular resistance, mainly due to^12^ abnormal proliferation of pulmonary vascular smooth muscle cells. Our previously published studies reported enhanced pulmonary vascular remodeling in the lungs from HIV infected opioids and/or cocaine users (HIV + IVDU) as depicted by abnormally thickened arterial smooth muscle layer^13^. This is interesting since HIV cannot productively infect pulmonary arterial smooth muscle cells (PASMCs), but instead is damaged due to the bystander effect of viral proteins released by infected macrophages and CD4^+^T-cells^12, 14^. The loss in the expression of bone morphogenetic protein receptors, one of the transforming growth factor β (TGFβ) superfamily of receptors, is a well-known cause of aggressive SMC proliferation. We previously reported severe down-modulation in the protein expression of BMPR-2, -1A and -1B and the corresponding down-stream signaling cascade in human PASMCs exposed to HIV-Tat, -Nef and -gp120 in the presence or absence of cocaine treatment^15^. Importantly, the lungs from HIV + IVDU that showed excessive pulmonary arterial SMC proliferation in the remodeled vessels had remarkable loss in BMPR-2, -1A and -1B expression^15^. Additionally, we recently published the BMPR dysfunction in PASMCs isolated from non-infectious HIV transgenic (Tg) rats exposed to cocaine^16^.

Many reports show that the failed anti-proliferative BMPR axis can stimulate the proliferative TGFβ1 arm of the TGFβ superfamily signaling, leading to excessive SMC proliferation and PAH^17–19^. Our earlier published results indicating higher Sma and Mad Related Family proteins (SMAD)4-SMAD2/3 complex and a significant reduced SMAD4-SMAD1/5/8 association in HIV-Tat and cocaine treated SMCs led us to delineate the pro-proliferative arm of the TGFβ super family signaling. Here, we report a remarkable increase in TGFβ1 ligand, TGFβ Receptor-1 and -2 and the corresponding downstream signaling in HIV-Tat and cocaine treated PASMCs. Taken together, we have established a definitive role of TGFβ signaling in HIV-PAH associated with IVDU.

## Results

### Increased expression of TGF-β receptor and its ligand during cocaine and HIV-Tat mediated pulmonary smooth muscle hyper-proliferation

**Figure 1:**
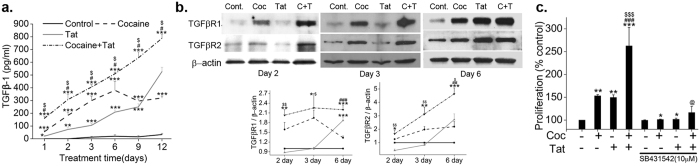
Cocaine and Tat induced increase in TGFβ ligand and receptor expression stimulates HPASMC proliferation. Quiescent HPASMCs were treated with cocaine (Coc) 1 µM and/or Tat (25ng/ml). (**a**) Cell supernatants collected from 1 to 12 days post-treatment were analyzed for TGFβ1 by ELISA. All values are mean ± SD of 3 independent experiments done in duplicates. *p ≤ 0.05 **p ≤ 0.01, ***p ≤ 0.001 compared to untreated control (Cont.), ^$^p ≤ 0.001 vs. Tat; ^#^p ≤ 0.001 vs. Coc. (**b**) Cells treated for 2, 3 or 6 days were lysed using RIPA buffer followed by western blot for TGFβR1 and 2. The graphs represent average densitometry of 3 independent experiments. (**c**) HPASMCs (3 × 10^3^/well) were seeded in 96 well plate, 48 h after which the medium was replaced with 0.1% serum containing medium followed by cocaine and/or Tat treatment for 2 days in presence or absence of TGFβR1 inhibitor: SB431542. MTS cell proliferation assay was then conducted. All values are mean ± SD of at least three independent experiments done in triplicates. *p ≤ 0.05 **p ≤ 0.01, ***p ≤ 0.001 compared to untreated control (Cont.), ^$^p ≤ 0.05, ^$$^p ≤ 0.01, ^$$$^p ≤ 0.001 compared to Tat; ^##^p ≤ 0.01, ^###^p ≤ 0.001 compared to Coc, @p ≤ 0.001 compared to cocaine and Tat combined treatment (C + T).

To understand the role of TGF-β signaling in cocaine and HIV-1 mediated smooth muscle hyperplasia^15^, we first investigated the levels of TGF-β1 ligand in the supernatants of cells treated with or without HIV-Tat and /or cocaine at various time intervals. As observed in Fig. 1a, single treatment with cocaine or Tat resulted in a significant increase in the secretion of TGF-β1 by HPASMCs compared to untreated control that progressively increased from early to later time points of treatment. However, maximum increase in TGF-β1 was seen on the combined treatment of cocaine and Tat treatment when compared with mono-treatments (Fig. 1a). Correspondingly, a significant increase in the protein expression of TGFβ receptor (TGFβR) -1 and (TGFβR)-2 was observed at day 2, 3 and 6 post-treatment of cocaine and Tat when compared with untreated control. This increase of TGFβR-1 and TGFβR-2 protein levels in cells treated with combined treatment was also significant when compared with Tat mono-treatment for 2–3 days or at all time intervals tested, respectively. However, when compared with mono-treatment of cocaine, the increase in TGFβR-1 and -2 on combined treatment was found to be significant only at 6 day post-treatment (Fig. 1b). As seen in Fig. 1c and Supplementary Figure S1, inhibition of TGFβ Receptor signaling using TGFβ-R1 active site inhibitor: SB431542 resulted in almost complete attenuation of cocaine and/or Tat mediated HPASMC hyper-proliferation. In addition, the reduction in the mono-treatments mediated increased proliferation was also observed on SB431542 pre-treatment.

### Augmentation of SMAD dependent TGFβ-Receptor downstream signaling in cocaine and HIV-Tat exposed smooth muscle cells

**Figure 2:**
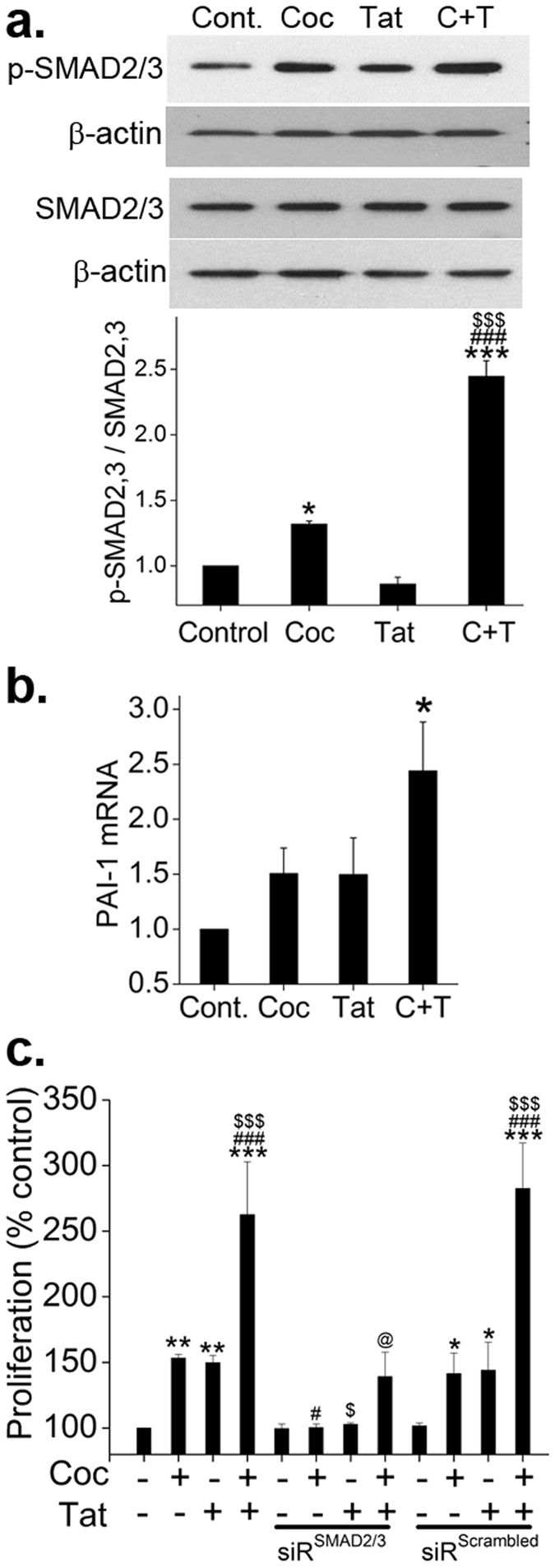
Augmented SMAD dependent TGFβ signaling in HPASMCs on exposure to combined treatment of cocaine and HIV-Tat. Quiescent HPASMCs were treated with cocaine 1 µM and/or Tat (25 ng/ml) for 2 days for western blot and RNA analysis. (**a**) Representative images of the Western blots probed using total and phosphorylated (p) - SMAD2/3. Graph represents average densitometry of 3 independent experiments. (**b**) Real time RT-PCR analysis of *PAI1* mRNA expression. (**c**) HPASMC (3 × 10^3^/well) were seeded in 96 well plate followed by either transfection with siRNA^SMAD2/3^ or siRNA^scrambled^. After 48 h, the medium was replaced with 0.1% serum containing medium followed by cocaine and/or Tat treatment for 2 days. MTS cell proliferation assay was then conducted. All values are mean ± SD of at least three independent experiments performed in triplicates. *p ≤ 0.05 **p ≤ 0.01, ***p ≤ 0.001 compared to untreated control (Cont.), ^$^p ≤ 0.05, ^$$$^p ≤ 0.001 compared to Tat; ^#^p ≤ 0.05, ^###^p ≤ 0.001 compared to cocaine (Coc), @p ≤ 0.001 compared to C + T.

We next investigated the levels of total and phosphorylated SMAD2/3, a component of TGFβR downstream signaling cascade. In confirmation to our earlier findings of increased SMAD2/3 and SMAD4 complex formation in cocaine and Tat treated HPASMCs^15^, we observed a significant increase in the levels of phosphorylated (p)-SMAD2/3 on combined cocaine and Tat treatment compared to single cocaine or Tat exposure or untreated cells (Fig. 2a) without any significant changes in total SMAD between all the groups. Also corresponding to our earlier published observations^15^ of increase in SMAD2/3 and SMAD 4 complex formation on cocaine treatment alone, we saw a significant increase in p-SMAD2/3 expression in cells treated with cocaine. Furthermore, we observed a significant increase in the expression of the downstream target gene of SMAD dependent TGFβ signaling, plasminogen activator inhibitor 1 (*PAI1*) in cocaine and Tat treated cells compared to mono- or untreated (Fig. 2b). We next tested whether this highly active SMAD2/3 signaling in cocaine and Tat treated cells contributed to the augmented proliferation by using siRNA against SMAD2/3. The transfection of cells with siRNA^SMAD2/3^ prevented the increase in proliferation of mono-treated HPASMC when compared with un-transfected or cells transfected with siRNA^scrambled^ (Fig. 2c). However, in case of combined cocaine-Tat treatment, the transfection of siRNA^SMAD2/3^ lowered the proliferation, but could not bring it back to the normal levels, therefore suggesting partial involvement of SMAD independent signaling in cocaine and Tat mediated augmentation of smooth muscle proliferation. For transfection efficiency see Supplementary Figure S2a.

### Alteration in TAK1 distribution across the proliferative and anti-proliferative arms of TGFβ signaling on cocaine and Tat exposure

**Figure 3:**
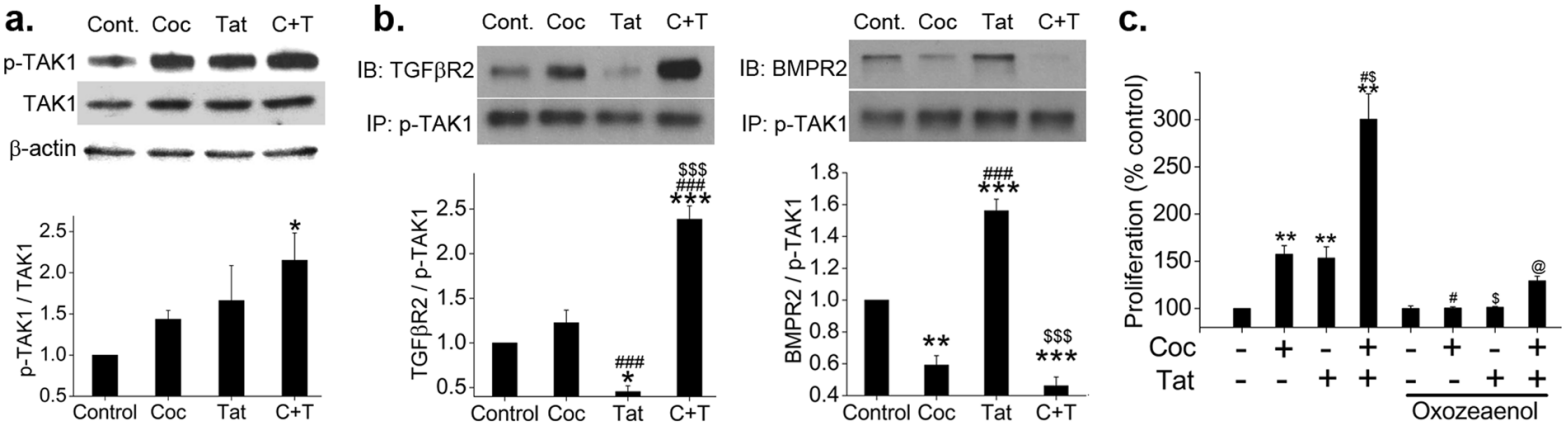
Cocaine-Tat mediated increase in TGFβR2 and TAK1 complex formation in hyperproliferative HPASMCs. Protein was extracted from quiescent HPASMCs treated with cocaine and/or Tat for 2 days followed by (**a**) Western blot for p-TAK1, TAK1 or **(b**) Immunoprecipitation (IP) using 50 µg of protein extract with phosphorylated TAK1 antibody bound to protein A/G agarose beads. Immunoblotting (IB) was later done with antibodies against TGFβR2 or BMPR2. Graphs represent densitometry analysis (mean ± SEM) of 3 independent experiments. (**c**) HPASMCs (3 × 10^3^/well) were seeded in 96 well plate. After 48 h the medium was replaced with 0.1% serum containing SMCM followed by cocaine and/or Tat treatment for 2 days in presence or absence of TAK1 inhibitor: oxozeaenol (10 µM). MTS cell proliferation assay was then conducted (mean ± SD). *p ≤ 0.05, **p ≤ 0.01, ***p ≤ 0.001 compared to untreated control, ^$^p ≤ 0.05, ^$$$^p ≤ 0.001 compared to Tat; ^#^p ≤ 0.05, ^###^p ≤ 0.001 vs. cocaine (Coc), ^@^p ≤ 0.001 compared to C + T.

The inability of siRNA^SMAD2/3^ to completely normalize the cocaine-Tat triggered HPASMC hyper-proliferation coupled with our earlier observation of impaired BMPR2 in cocaine-Tat treated HPASMCs ^15^ led us to investigate the role of TGFβ-associated kinase 1 (TAK1) that is known to promote proliferative TGFβ signaling in case of dysfunctional anti-proliferative BMPR-2 signaling^19^. We observed an increase in the levels of activated phosphorylated (p)-TAK1 as well as total TAK1 in the cells exposed to either combined or mono-treatment when compared with untreated cells. However after normalizing the expression of p-TAK1 with total TAK1, the significant increase in active TAK1 was observed only with combined treatment (Fig. 3a). Next we looked at the association of TAK1 with TGFβ-Receptor and BMPR-2 by performing immune-precipitation of p-TAK1 followed by western blot analysis for TGFβ-R2 and BMPR-2. As shown in Fig. 3b, we saw a significant increase in TGFβR2-p-TAK1 complex formation in cocaine and Tat treated cells compared to monotreatments or untreated control. Interestingly, a significant reduction in the TGFβR2-p-TAK1 complex was observed in cells treated with Tat alone. Conversely, a significant decrease in BMPR2-p-TAK1 complex was observed on combined treatment and a significant increase in this complex formation was found in cells treated with Tat alone. Monotreatment with cocaine alone resulted in statistically insignificant increase in TGFβR2-p-TAK1 complex with a significant decrease in BMPR2-p-TAK1 complex formation. Next, we tested if this TAK1 activation plays a role in cocaine-Tat mediated HPASMC proliferation. As expected, inhibiting TAK1 using oxozeaenol (Fig. 3c) or siRNA^TAK1^ (Supplementary Figure S3c) caused significant reduction in cocaine and Tat mediated increased HPASMC proliferation. As in case of siRNA^SMAD2/3^ (Fig. 2c), inhibition of SMAD independent TAK1 signaling by siRNA^TAK1^ or oxazeaenol could not completely reduce the combined cocaine and Tat mediated HPASMC hyper-proliferation. The representative western blots indicating the transfection efficiency are shown in Supplementary Figure S2b.

### Increased activation of MKK4-JNK1 axis but decreased activation of MKK3/6-p38MAPK axis on cocaine and Tat exposure

**Figure 4:**
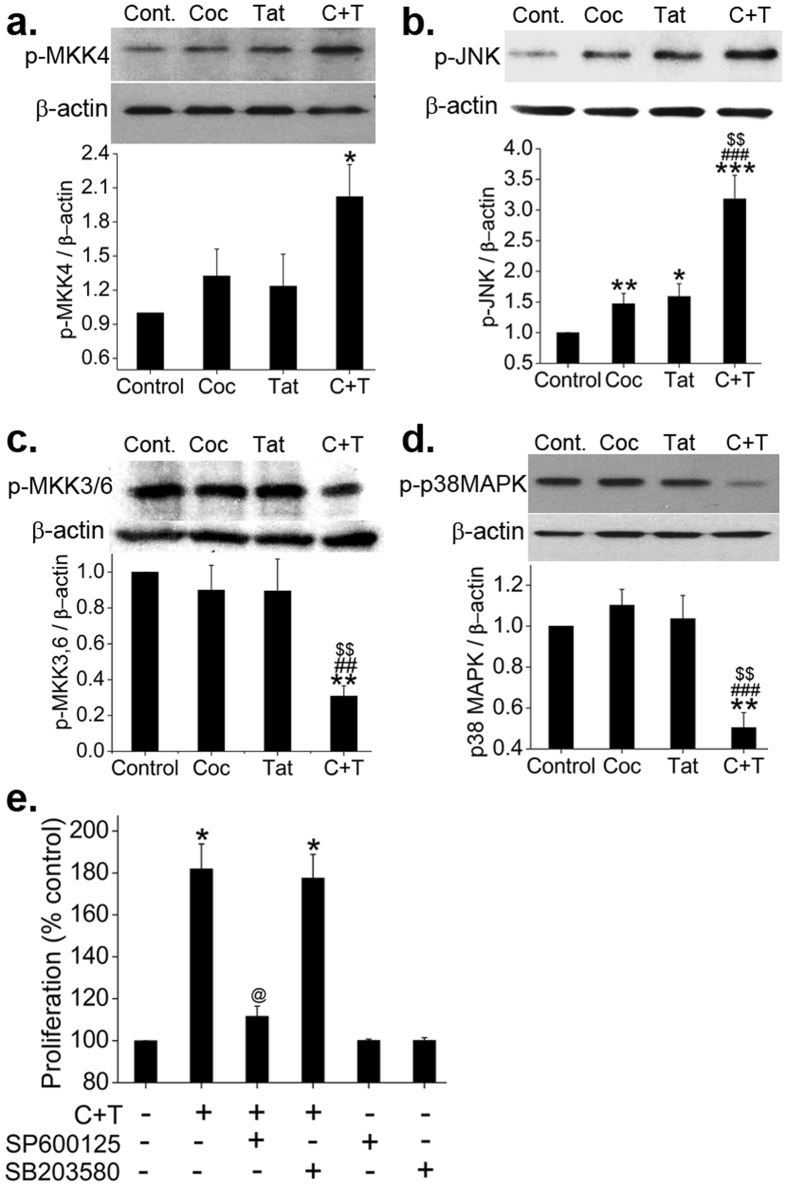
Activation of SMAD independent TAK1 dependent MKK4-JNK axis in Tat and cocaine treated HPASMCs. (**a–d**) Quiescent HPASMCs were treated with cocaine (Coc) and/or Tat for 2 days followed by protein extraction and western blot. Graphs represent average densitometry of 3 independent experiments (mean ± SEM). (**e**) MTS cell proliferation assay was conducted on 48 h cocaine and/or Tat treated HPASMCs plated as described in Figs 1 and 2 in presence or absence of JNK inhibitor SP600125 or p38MAPK inhibitor SB203580. *p ≤ 0.05,**p ≤ 0.01,***p ≤ 0.001 compared to control, ^$$^p ≤ 0.01 compared to Tat; ^##^p ≤ 0.01, ^###^p ≤ 0.001 compared to Coc, ^@^p ≤ 0.001 compared to C + T.

We then continued to explore the downstream signaling cascades known to be regulated by TAK1, the SMAD independent arm of TGFβ signaling. We observed a significant increase in p-MKK4 expression and also in its corresponding downstream molecule p-JNK1 in cocaine and Tat treated cells compared to untreated control (Fig. 4a and b). Interestingly, the phosphorylated active forms of MKK3/6, and p38MAPK that are also downstream of TAK1 signaling showed a significant reduction in dual treated cells compared to untreated, as shown in Fig. 4c,d and Supplementary Figure S4. Further using small molecule inhibitors of JNK1 and p38MAPK, we also observed that the augmented proliferation of HPASMCs with dual treatment of cocaine and Tat is dependent on JNK1 activation but independent of p38MAPK activation as presented in Fig. 4e.

### TAK1 independent regulation of MKK3/6 and p38MAPK in cocaine and Tat treated HPASMCs

**Figure 5:**
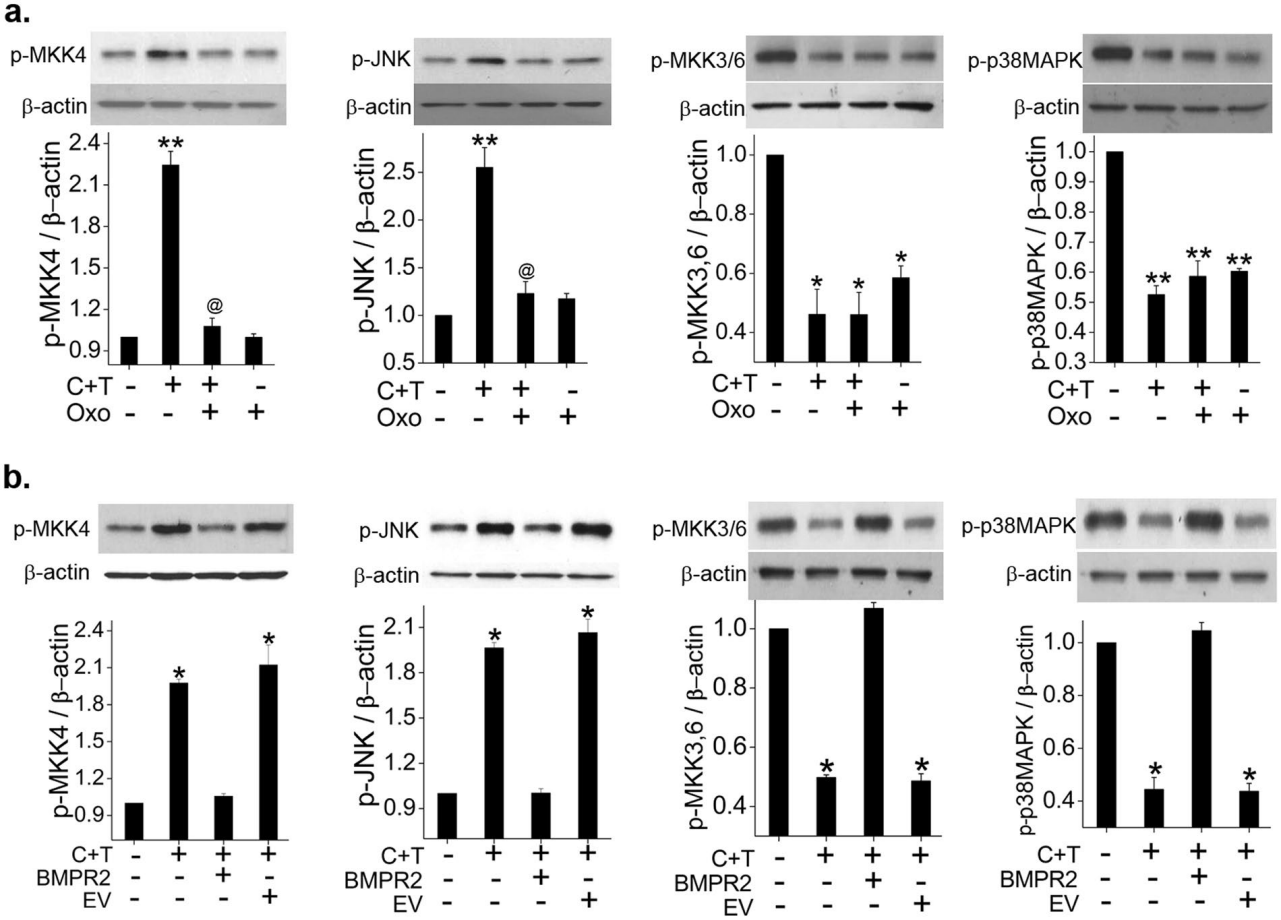
TAK1 independent downregulation of p38MAPK on cocaine and Tat treatment. (**a**) Quiescent HPASMCs were treated with cocaine (C) and/or Tat (T) for 2 days with or without 10 μM oxozeaenol (Oxo) pretreatment followed by protein extraction and western blot. (**b**) HPASMCs were transfected with either PCMV6-XL5-BMPR2 plasmid or empty PCMV6-XL5 vector (EV) and after 2 days post-transfection cells were serum starved for 48 h followed by 48 h cocaine and Tat (C + T) treatment for western blot analysis. Graphs represent average densitometry of 3 independent experiments (mean ± SEM). *p ≤ 0.05,**p ≤ 0.01 compared to control, ^@^p < 0.01 compared to C + T.

After observing an unexpected reduction in p-MKK3/6 and p-p38MAPK expression in cocaine and Tat treated cells, we examined if this occurrence was independent of TAK1. As illustrated in Fig. 5a, inhibition of TAK1 using oxozeaenol resulted in complete knockdown of the cocaine and Tat mediated increase in the levels of p-MKK4 and p-JNK1. However, pre-treatment with oxozeaenol had no effect on the cocaine and Tat mediated significant reduction in the activation of MKK3/6 and p38MAPK (Fig. 5a). These findings along with the results illustrated in Fig. 4(c and d) confirm that the cocaine and Tat mediated alterations in the MKK3/6 and p38MAPK axis is independent of TAK1 activation in HPASMCs. We also observed that the cells treated with only oxozeaenol showed a significantly reduced p-MKK3/6 and p-p38MAPK expression without any toxic effect on cells (Fig. 5a). These findings were further confirmed using siRNA^TAK1^ (Supplementary Figure S3a and b).

Given that MKK3/6 and p38MAPK are also known to be activated by BMPR signaling, and we earlier reported significant attenuation of BMPR2 in cocaine and Tat treated HPASMCs^15^, we next examined the effect of BMPR2 overexpression on MKK4-JNK1 and MKK3/6-p38MAPK axis. As expected, no increase in the activation of MKK4-JNK1 axis was observed on cocaine-Tat treatment of HPASMCs overexpressing BMPR2 (Fig. 5b). Conversely, over expression of BMPR2 prevented the cocaine and Tat mediated reduction in levels of pMKK3/6 and p-p38MAPK (Fig. 5b).

### TGFβR activation in pulmonary arterial smooth muscle cells from HIV-Tg rats and in lungs from HIV infected individuals on exposure to cocaine

**Figure 6:**
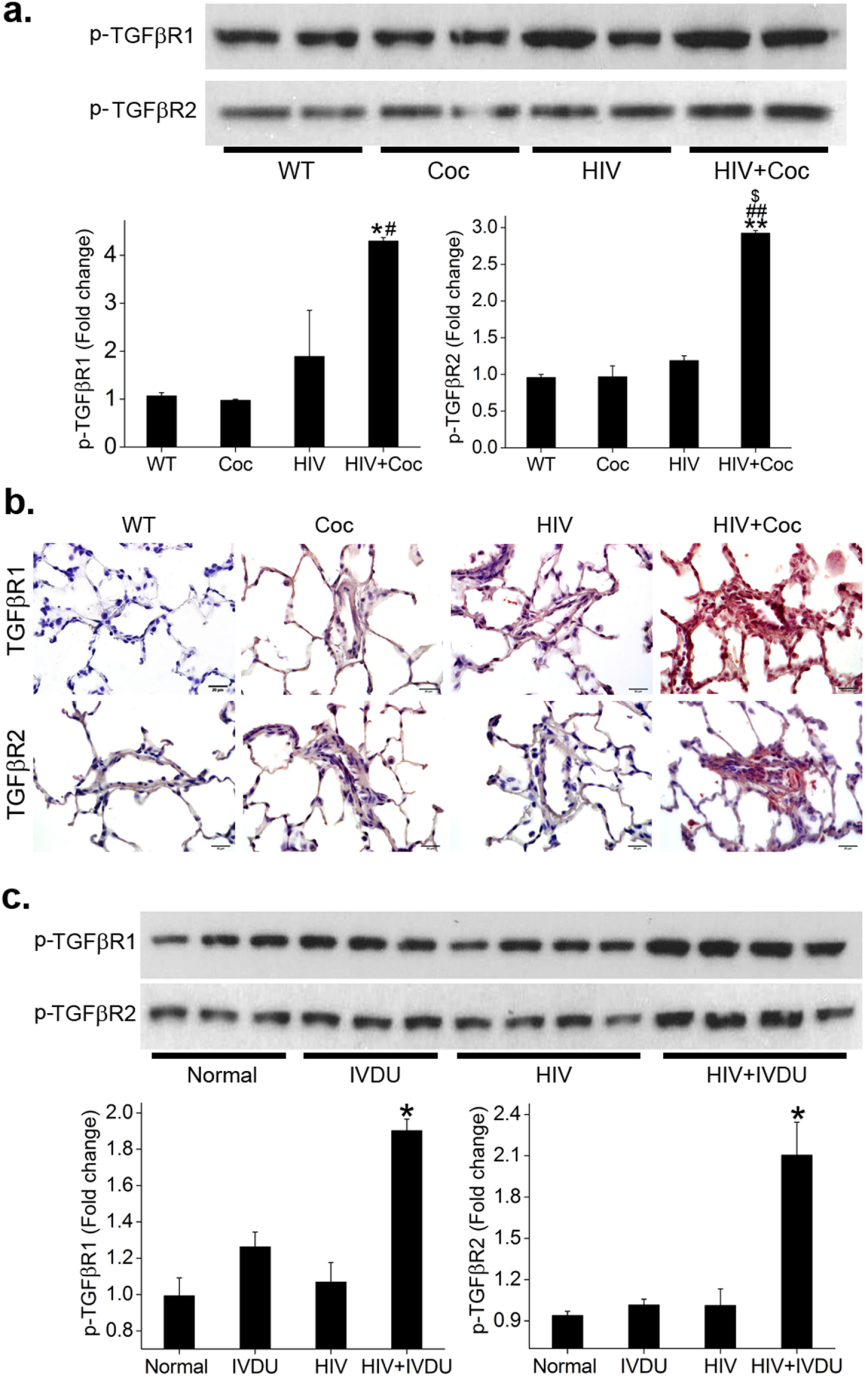
Increase in the activation of TGFβR-1 and -2 in lungs from cocaine exposed HIV-transgenic rats with PAH and from HIV infected opioid/cocaine abusers. (**a**) Rat PASMCs isolated from HIV-Tg or wild-type (WT) rats treated with or without cocaine were lysed using RIPA buffer followed by immunoprecipitation (IP) using 2 µg of TGFβR-1 or -2 antibody. Immunobloting (IB) was next perfomed using p-TGFβR-1 or -2 respectively. (**b**) Paraffin embedded lung sections from various goup of rats were immuno-stained using primary antibody against total TGFβR-1 and -2. Scale: 20 µm. (**c**) Total proetin extract of frozen human lung tissues from normal, IVDU, HIV and HIV + IVDU was used for immunoprecipitation and western blot as mentioned for rat PASMCs. Mean ± SEM. *p ≤ 0.05,**p ≤ 0.01 compared to WT/normal, ^#^p < 0.05, ^##^p < 0.01 compared to cocaine, ^$^compared to HIV.

We recently reported elevated mean pulmonary arterial pressure (mPAP) and right ventricle systolic pressure (RVSP) in HIV-Tg rats exposed to cocaine with concomitant down-regulation of BMPR expression and downstream signaling in the hyper proliferative arterial smooth muscle cells isolated from these cocaine injected HIV-Tg rats^16^. In this study we used pulmonary arterial smooth muscle cells from these HIV Tg rats to analyze the status of TGFβ receptors in the presence or absence of cocaine. As shown in the western blot images in Fig. 6a, there was a significant increase in the levels of phosphorylated (p)-TGFβR-1 and -2, in the rat pulmonary arterial SMC (RPASMCs) isolated from HIV-Tg rats exposed to cocaine when compared with RPASMCs isolated from untreated HIV-Tg rats or from wild-type (WT) rats treated with or without cocaine. Similarly, immunohistochemistry analysis of rat lung sections showed an overall increase in the expression of TGFβR-1 and -2 in rats from HIV+ cocaine group compared to rat sections from HIV or WT+/−cocaine groups. Particularly, there was a notable increase in the expression of these two receptors in the remodeled vessels of the HIV+ cocaine rats as represented in Fig. 6b. Corresponding to these *in-vivo* murine findings, we also observed a remarkable increase in the expression of p-TGFβR-1 and -2 in the total lung extracts from HIV infected IVDUs (cociane+/−opioid users) (HIV + IVDU) when compared with lung extracts from HIV-infected non drug users (HIV), un-infected IVDUs or normal controls (Fig. 6c). Moderate increase in TGFβR2-p-TAK1 complex was also observed in total lung extract from HIV + IVDUs compared with normal controls (Supplementary Figure S5). These data strongly suggest an important role of TGFβR dysregulation in the pulmonary vascular remodeling associated with HIV infection and opioid use.

## Discussion

HIV related PAH is a serious problem considering the unresponsiveness of the affected patients to vasodilators or supplemental oxygen^20^. Importantly, drug abuse of opioids/stimulants such as cocaine can further increase the chances of PAH development in HIV infected individuals^8^. Our group has been studying this salient relation between HIV infection and the use of illicit drugs for the past several years^13, 15, 16, 21–23^ and one of the many noteworthy findings we reported earlier is the severe attenuation of BMPR mediated signaling in pulmonary SMCs exposed to HIV-proteins such as Tat in combination with cocaine^15^. In addition, enhanced levels of phosphorylated SMAD2/3 complexed with SMAD4 are observed on combined treatment, suggesting activation of an alternative proliferative TGF-β receptor pathway^15^. In extension to these previous findings, our current study demonstrates enhanced protein expression of TGFβR1, TGFβR2 and significant increase in the activation of SMAD dependent and TAK1 mediated SMAD independent downstream signaling cascades in response to the combined treatment of cocaine and HIV-Tat compared to either treatment alone. The concentrations used for cocaine and Tat are based on previous published findings where while testing different concentrations, combination of 1 µM cocaine and 25ng/ml HIV-Tat gave the maximum synergistic increase in the proliferation of smooth muscle cells^13^.

Various published findings have earlier reported abnormal hyperactive TGFβ signaling in case of dysfunctional BMPR2 expression in pulmonary smooth muscle cells^18, 19, 24, 25^. Very recently, Harper *et al*.^26^ and Feng *et al*.^27^ provided an *in-vitro* evidence of noteworthy prevention of TGFβ mediated SMAD1/5/8 phosphorylation inhbition and a corresponding significant decrease in TGFβ stimulated SMAD2/3 phosphorylation in HPASMCs on BMPR2 overexpression, re-establishing the normal function of TGFβ in the setting of optimal BMPR2 expression. Likewise in our current study we demonstrate an increase in TGFβ1 ligand and a corresponding increase in the activation of TGFβRs in cocaine and Tat treated HPASMCs with dysfunctional BMPR signaling. In our earlier report where we observed a reduced formation of SMAD1/5/8 and SMAD4 complex in HIV-Tat and cocaine exposed HPASMCs, a significant increase in SMAD2/3 and SMAD4 complex^15^ was also found. Furthermore, our current data demonstrating a significant increase in the downstream SMAD 2/3 activation in cocaine and Tat treated HPASMCs but reduction in SMAD2/3 activation in cells treated with Tat alone correlate with our earlier published findings of reduced SMAD1/5/8 activation on combined treatment and increased activation in Tat treated cells^15^. Correspondingly, we also now observed a significant increase in the mRNA expression of TGFβR-SMAD2/3 donwstream target: *PAI1* on combined treatment. Consistent with our study, Upton *et al*.^18^ and Davies *et al*.^17^ have shown significant increased expression of *PAI1* in TGFβ stimulated HPASMCs compared to control.

Further corroborating these data, we observed complete normalization of cocaine-Tat stimulated HPASMCs proliferation in presence of TGFβR1 inhibitor: SB431542. However, in our studies silencing of SMAD2/3 in cocaine and Tat treated HPASMCs could not completely bring back the cell proliferation to the levels of untreated control cells as in case of mono-treatments. This clearly indicates the partial involvement of additional signaling pathway(s) downstream of TGFβRs in cocaine and Tat mediated HPASMCs proliferation. Numerous reports have shown the involvement of non-canonical SMAD-independent TGFβR signaling in the regulation of cell proliferation^28^. Nasim *et al*.^19^ reports that when BMP signaling is attenuated, the TAK1, that competes between BMP and TGFβ arms of TGFβ superfamily signaling, is entirely available for TGFβ receptor complex and this imbalance leads to over-activation of non-SMAD pathway resulting in hyper-proliferation of PASMCs. Interestingly, we found a significant increase in the formation of p-TAK1-TGFβR2 complex in HPASMCs exposed to combined treatment of cocaine and Tat. Whereas p-TAK1-BMPR2 complex was found to be significanty diminished in these cells owing to the loss of BMPR2 protein on cocaine and Tat treatment as reported by us previously^15^. Concomitant to these findings we observed a significant increase in MKK4-JNK axis that is known to be downstream of TAK1^28^ in dual treated cells. In addition, inhibition of TAK1 and JNK using pharmacological inhitors could significantly prevent the cocaine and Tat mediated augmentation in cell proliferation, therefore proving the importance of TAK-MKK4-JNK axis. Overall these findings suggest that the reduction in the expression of BMPR2 receptors^15^ in cocaine and Tat treated HPASMCs results in the availability of TAK1 for binding to TGFβ receptor complex leading to the activation of pro-proliferative SMAD independent signaling.

However, MKK3/6-p38MAPK axis that is also known to be activated by TAK1 was down-regulated in dual treated cells. This along with the findings demonstrating no change in the activation of MKK3/6 and p38MAPK in cocaine-Tat treated HPASMCs on inhibiting TAK1 confirmed that TAK1 activation was not involved in the regulation of MKK3/6-p38MAPK axis in cocaine-Tat treated cells. Also notably, inhibiting p38MAPK by pharmacological inhibitor did not change the cocaine-Tat stimulated HPASMCs hyper proliferation. These findings relate to the report by Liu *et al*. showing increased BMPR2, p-p38MAPK but no change in p-JNK expression in BMP2 treated mouse embryonic fibroblasts^29^. Furthermore, the prevention of cocaine and Tat mediated reduction in the level of p-p38MAPK in cells overexpressing BMPR2 confirmed the importance of p38MAPK activation in the downstream antiproliferative response of BMPR signaling as reported by Yuan *et al*.^30^. All these findings indicate that the attenuation in the expression of BMPRs in HPASMCs in response to dual treatment with cocaine and Tat^15^ results in reduced activation of p38MAPK activation independent of TAK1 activation in response to TGFβ receptor signaling.

The BMP-TGF signaling dysfunction observed in HPASMCs on exposure to cocaine and Tat was also reflected in PASMCs isolated from cocaine injected HIV-Tg rats and in whole lung extracts from HIV infected cocaine and/or opioid users. To the best of our knowledge, this is the first report on significantly increased expression of TGFβR in the animal model of HRPAH exposed to cocaine as well as in HIV infected IVDUs.

In conclusion, we demonstrate involvement of both SMAD dependent and SMAD indepedent (TAK1-MKK4-JNK) TGF-β signaling in cocaine and Tat mediated SMC hyperplasia. However, MKK3/6-p38MAPK axis was found to be reduced on combined treatment independent of TAK1 activation in the setting of down-modulation of BMPR axis. Beraprost sodium is now being used for the treatment of pulmonary hypertension that acts by inhibiting overactive TGFβ signaling by reducing SMAD3 and p38MAPK^24^. Our current results suggest potential value in the consideration of the development of alternative strategies for combating HRPAH that may not be associated with p38MAPK activation in hyper-proliferative smooth muscle cells with dysfunctional BMPR signaling.

## Materials and Methods

### Cell Culture and Treatments

Primary human pulmonary arterial smooth muscle cells (HPASMCs) purchased from ScienCell research laboratories (Carlsbad, CA) were cultured in 2% fetal bovine serum (FBS), smooth muscle cell growth supplements and penicillin/streptomycin containing smooth muscle cell media (SMCM) (ScienCell research laboratories). Cells were treated with cocaine (1 µM) and/or recombinant HIV-Tat (25 ng/ml) (ProspecTany laboratories, Israel) for various time points in SMCM without FBS and growth factors after 48 hours of serum starvation at 80% confluency. The concentrations of cocaine and Tat were based on our previously published findings^13^.

### ELISA

Confluent HPASMCs grown on 96 well plates were treated with or without cocaine and Tat in serum free SMCM every day and cell supernatant was collected at intervals from 1 to 12 days followed by ELISA for TGFβ-1 ligand (R&D Systems, Minneapolis, MN).

### Western Blot analysis and co-immunoprecipitation

HPASMCs serum starved in SMCM without growth factors for 48 h were treated for 2, 3 or 6 days with cocaine in presence or absence of Tat. Cells were lysed with RIPA buffer (Santa Cruz Biotechnology, Dallas, TX) followed by western blot for antibodies against TGFβRI, TGFβRII, BMPR-2, total and phosphorylated (p)-SMAD2/3, TAK1 (Santa Cruz Biotechnology, Dallas, TX), p-TAK1, p-MKK3/6, p-MKK4, p-p38MAPK, p-JNK1 (Cell signaling, Beverly, MA), p-TGFβRI (Abcam, Cambridge, MA), p-TGFβRII (Biorbyt, San Francisco, CA) and β-actin (Sigma-Aldrich, St. Louis, MO). For the detection of protein expression, enhanced chemiluminescence system (Thermo Scientific, Rockford, Illinois) was used after secondary probing with HRP-conjugated anti-mouse or anti-rabbit antibodies (Millipore, Billerica, MA). The density of bands was analyzed using the NIH ImageJ software. For co-immunoprecipitation total cellular protein (50 µg) was incubated at 4 °C, overnight with 1 µg of anti-p-TAK1 followed by immunoprecipitation using protein A/G agarose beads (Thermo Scientific) as per manufacturer’s instructions. Western blot was then performed to compare BMPR2-p-TAK1 complex with TGFβR2-p-TAK1 complex with 25 μg of precipitated protein using antibodies against BMPR2 and TGFβR2.

### Transfection with BMPR2 over-expression plasmid

After 2 days of transfecting HPASMC with either PCMV6-XL5-BMPR2 plasmid or empty PCMV6-XL5 vector (Origene Technologies, Inc., Rockville, MD) as described previously^15^, cells were serum starved for 48 h followed by cocaine and Tat treatment. Cells were lysed after 2 days and assayed for p-MKK3/6, p-p38MAPK, p-MKK4 and p-JNK expression by western blot.

### Cell proliferation assay

HPASMCs seeded and starved in 0.1% FBS containing SMCM in 96 well plate as described previously^15^ were pre-treated with either SB431542 or oxozeaenol 20 min before cocaine and/or Tat for 6 days or alternatively with JNK1inhibitor: SP600125 or p38MAPK inhibitor: SB203580 before cocaine-Tat treatment for 2 days followed by CellTiter 96^®^ Aqueous One Solution Cell Proliferation Assay (Promega, Madison, WI) according to the manufacturer’s instructions. The 10 µM concentration of various inhibitors used was based on published articles that reported their use on smooth muscle cell cultures^19, 31–33^. In another set of experiments, HPASMCs transiently transfected with siRNA against SMAD-2, SMAD-3, TAK1 or with scrambled siRNA as described previously^23^ were treated with cocaine and/or Tat for 6 days followed by cell proliferation assay.

### RT-PCR

Confluent HPASMCs were treated with cocaine and/or Tat for 2 days followed by RNA extraction using Trizol reagent (Thermo Scientific, Waltham, MA) and cDNA preparation using miScript cDNA synthesis kit (BIO-RAD, Hercules, CA). Quantitative RT-PCR was then performed for measuring plasminogen activator inhibitor (*PAI1*) expression using SYBR green reagent (Applied Biosystems, Foster City, CA) as mentioned in our previous publications^15^.

### HIV-1 Transgenic and wild type rats

Four month old male Fischer HIV-1 Tg rats (n = 3–5/group) expressing HIV-1 proteins in lymphocytes and monocytes and wild type (WT) Fischer334 rats were purchased from ENVIGO (Indianapolis, Indiana). HIV-Tg rats were administered 40 mg/kg body weight of cocaine (HIV + cocaine group) or saline (HIV group) intraperitoneally, once daily for 21days^16^. WT rats were also injected with either cocaine (cocaine group) or saline (control group). The animals were housed at the University of Kansas Medical Center as per the National Institutes of Health (NIH) Guide for the Care and Use of Laboratory Animals. Hemodynamic measurements on these rats were reported in our recent publication^16^. Smooth muscle cells isolated from pulmonary arteries dissected from these rats were cultured in rat smooth muscle cell medium and used up to passage 4 to analyze the expression of phosphorylated (p)-TGFβR-1(Abcam) or p-TGFβR-2 (Biorbyt, San Francisco, CA) by immunoprecipitation of the extracted protein with the corresponding total TGFβR antibodies against (Santa Cruz Biotechnology, Dallas, TX) followed by western blot as described previously^16^. Immunohistochemistry for TGFβR-1 and -2 was performed on paraffin embedded lung sections.

### Human lung tissues

For *ex-vivo* analysis of TGFβR expression in humans we used archival lungs from de-identified HIV-infected IV opioids and/or cocaine abusers that demonstrated enhanced pulmonary vascular remodeling compared to lungs from HIV non-drug abusers or un-infected IVDUs^13^. The four groups of frozen human lung tissues, HIV + IVDU (n = 4), HIV (n = 4), IVDU (n = 3) were obtained from the Manhattan HIV Brain Bank (R24MH59724; U01MH083501; New York, NY) and un-infected non-IVDU controls (Normal, n = 3) were obtained from National Disease Research Interchange (NDRI, Philadelphia, PA). The details regarding the clinical, pathological and demographic characteristics of these human subjects are previously reported by us^13^.

### Statistical Analysis

One-way analysis of variance with a *post hoc* Bonferroni test for multiple comparisons was performed using GraphPad Prism software (GraphPad Software Inc., La Jolla, CA). The results are represented statistically significant when the Bonferroni corrected P values were less than 0.05. Due to the limited sample size in case of the human lung tissues, the non-parametric Mann Whitney test was used at 0.05 confidence level.

## Electronic supplementary material

Supplementary Figures.
