# Supplementary material for: Hyperactive TGF-β Signaling in Smooth Muscle Cells Exposed to HIV-protein(s) and Cocaine: Role in Pulmonary Vasculopathy
Source: Sci Rep. 2017 Sep 5;7:10433. doi: 10.1038/s41598-017-10438-3 (PMC5585314; doi:10.1038/s41598-017-10438-3)
Supplement: Supplementary file 1 — Supplementary Figures. [file 41598_2017_10438_MOESM1_ESM.doc]

**SUPPLEMENTAL METERIAL**

**Hyperactive TGF-β Signaling in Smooth Muscle Cells Exposed to HIV-protein(s) and Cocaine: Role in Pulmonary Vasculopathy.**

Pranjali Dalvi^1^, Himanshu Sharma^1^, Tomara Konstantinova^1^, Miles Sanderson^1^, Amy O’Brien-Ladner^1^, Navneet K. Dhillon^1,2^.

^1^Division of Pulmonary and Critical Care Medicine, Department of Medicine, ^2^Department of Molecular & Integrative Physiology, University of Kansas Medical Center, Kansas City, Kansas , USA.

Correspondence and requests for reprints should be addressed to Navneet K. Dhillon, Division of Pulmonary and Critical Care Medicine, Department of Medicine, Mail Stop 3007, University of Kansas Medical Center, 3901 Rainbow Blvd, Kansas City, KS 66160, Tel: (913) 945-6018, Fax: (913) 588-4098, Email: ndhillon@kumc.edu

**Supplementary Figure S1:**

**
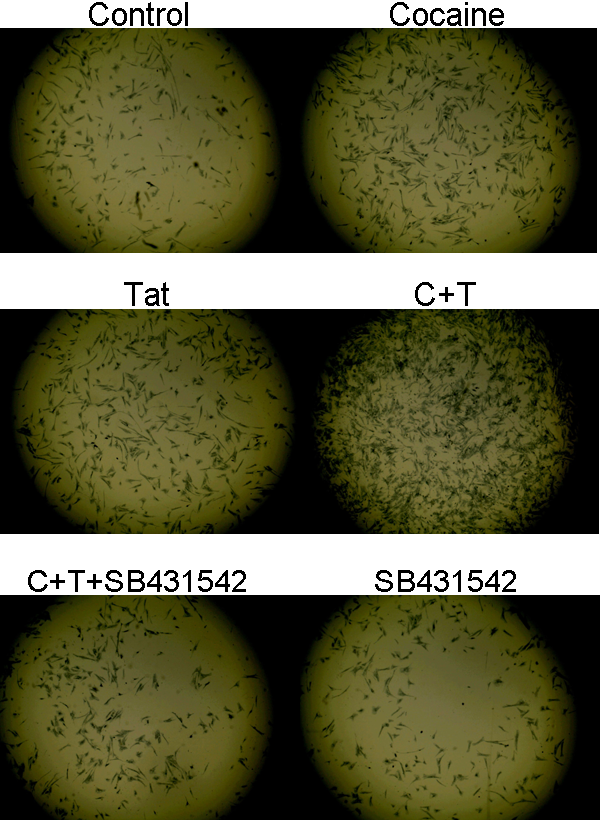
**

**Increase in cell proliferation on exposure to cocaine and HIV-Tat.** HPASMC (3X10^3^/well) grown in 96 well plate were starved in 0.1% serum containing smooth muscle cell medium for 2 days after 48h of seeding. The medium was then replaced and cells were treated with TGFβR1 inhibitor: SB431542 followed by cocaine and/or Tat for 2 days. Images were captured under light microscope at 4X magnification after MTS analysis.

**Supplementary Figure S2:**


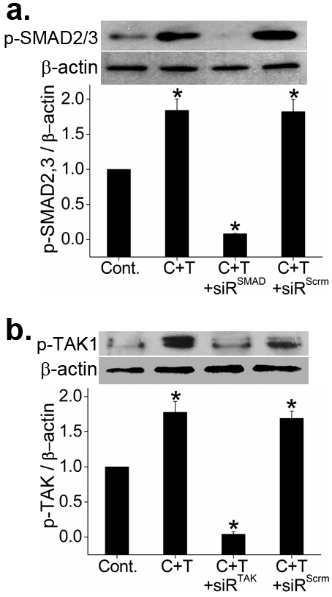


**Evaluation of transfection efficiency of siRNA^SMAD2,3^ and siRNA^TAK1^.** HPASMC were transfected with the respective siRNAs as mentioned in the figure using HiPerfect reagent followed by cocaine and Tat treatment for 6d after which protein was extracted from the cells and western blot for activated (phosphorylated: p) SMAD2/3 **(a)** or TAK1 **(b)** respectively was performed. All values are mean +SD of two independent experiments. * p<0.001 compared to control. Scrm: Scrambled.

**Supplementary Figure S3:**

**
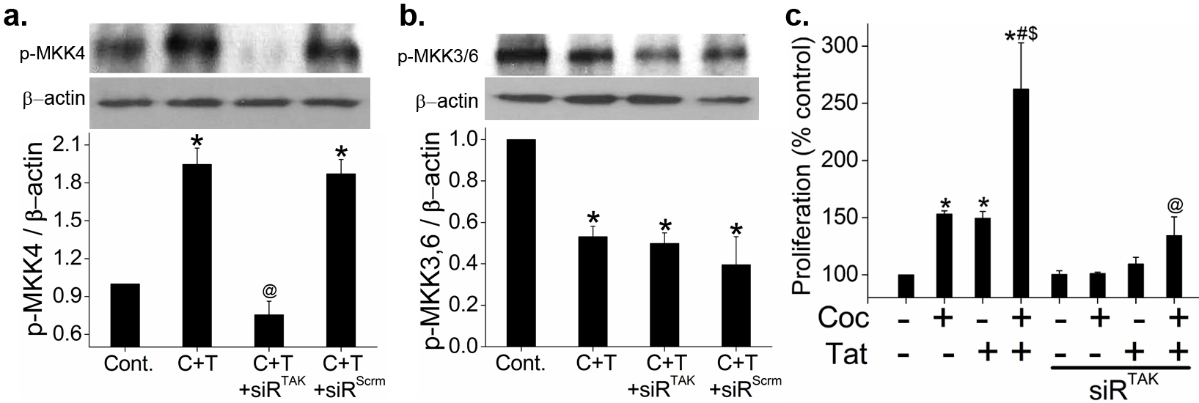
**

**Decrease in MKK3/6-p38MAPK activation in cocaine-Tat treated HPASMC is independent of TAK1. a and b)** HPASMC transfected with siRNA^TAK1^, treated with cocaine and Tat (C+T) for 2 days were lysed using RIPA buffer followed by western blot. Cocaine-Tat treated cells transfected with siRNA^Scrambled^ (Scrm) were used as control. Graphs represent the average densitometry of three independent experiments, mean +SEM. **c)** HPASMC transfected with siRNA^TAK1^ on 96 well plate and treated with cocaine and/or Tat for 6 days were assayed for proliferation using MTS reagent. Mean +SEM of 3 independent experiments done in triplicates. *p<0.05 compared to control, #p<0.05 compared to cocaine, $p<0.05 compared to Tat, @p<0.05 compared to C+T.

**Supplementary Figure S4:**

**
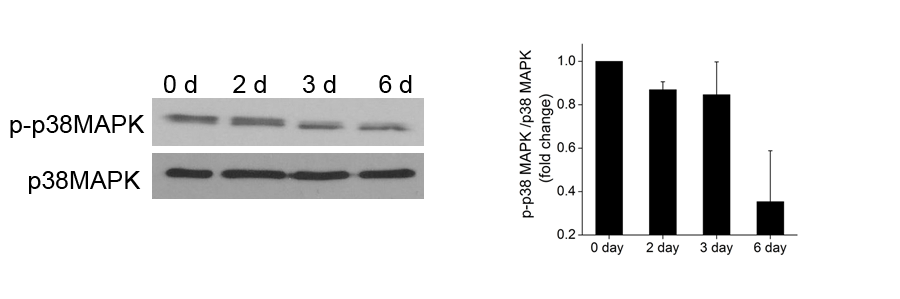
**

**Lack of increase in p-38MAPK phosphorylation (p) in HPASMCs treated with cocaine and Tat.** Cells were treated with cocaine and Tat daily followed by protein extraction for western blot analysis at indicated time intervals. Graph represents the densitometry analysis of 2 independent experiments. +SEM.

**Supplementary Figure S5:**


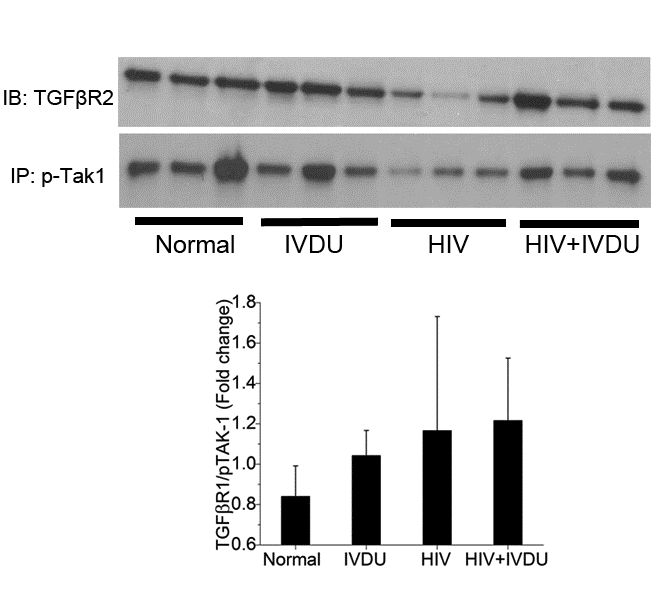


**Level of TGFβR2-p-TAK1 complexes in lungs from HIV and /or IVDUs.** Protein from frozen human lung tissues was extracted using RIPA buffer. Samples were immunoprecipitated using p-TAK1 (1µg) antibody followed by western blotting for TGFβR2. Graph represents the densitometry analysis of n=3 per group ( average + SEM).
